# Artificial Intelligence in Functional Food Ingredient Discovery and Characterisation: A Focus on Bioactive Plant and Food Peptides

**DOI:** 10.3389/fgene.2021.768979

**Published:** 2021-11-19

**Authors:** Aoife Doherty, Audrey Wall, Nora Khaldi, Martin Kussmann

**Affiliations:** Nuritas Ltd., Dublin, Ireland

**Keywords:** bioactive, food ingredient, functional food, artificial intelligence, characterisation, mass spectrometry, high-throughput screening, discovery

## Abstract

Scientific research consistently demonstrates that diseases may be delayed, treated, or even prevented and, thereby, health may be maintained with health-promoting functional food ingredients (FFIs). Consumers are increasingly demanding sound information about food, nutrition, nutrients, and their associated health benefits. Consequently, a nutrition industry is being formed around natural foods and FFIs, the economic growth of which is increasingly driven by consumer decisions. Information technology, in particular artificial intelligence (AI), is primed to vastly expand the pool of characterised and annotated FFIs available to consumers, by systematically discovering and characterising natural, efficacious, and safe bioactive ingredients (bioactives) that address specific health needs. However, FFI-producing companies are lagging in adopting AI technology for their ingredient development pipelines for several reasons, resulting in a lack of efficient means for large-scale and high-throughput molecular and functional ingredient characterisation. The arrival of the AI-led technological revolution allows for the comprehensive characterisation and understanding of the universe of FFI molecules, enabling the mining of the food and natural product space in an unprecedented manner. In turn, this expansion of bioactives dramatically increases the repertoire of FFIs available to the consumer, ultimately resulting in bioactives being specifically developed to target unmet health needs.

## Functional Food Ingredients

### Consumer Demand for Science-Backed Healthy Ingredients and Natural Foods

With the recent exponential surge in information technology advancements, today’s consumers demand instant access to deeper product understanding unlike any generation before. For the food and nutrition market, this means that the next wave of economic growth will be driven by nutrition-based technology that empowers consumers to take better control of their own health by accessing validated information about beneficial effects of natural ingredients and supplements for various conditions (Doub et al., 2015; Chen et al., 2018; Littlejohn et al., 2018). Market trends are demonstrating that consumers are 1) increasingly expecting robust scientific evidence for nutritional claims (Talati et al., 2016; Lin, Shih, and Lin 2017); 2) educating themselves to understand the meaning and validity of Functional Food Ingredients (FFIs) and their associated health claims (A. Zhang et al., 2020; Hartmann et al., 2018); 3) becoming confident to express their opinion regarding the safety and benefits of food products (Savchenko et al., 2019); and 4) identifying which food characteristics, such as food naturalness and sustainability, are important to them (Román, Sánchez-Siles, and Siegrist 2017). In particular, there is a need for standardisation and transparency when it comes to health claims for additives produced by food and nutrition companies. For example, E-numbers (codes used to describe substances used as food additives throughout the European Union) are a set of standardised codes brought in to ensure uniformity in additive description and as studies are showing consumers prefer simpler health claims (Lynam, 2011), an approach should be developed to standardise health claims, similar to E-numbers, which is already an active area of research (Gallagher et al., 2011). Ultimately, consumers are currently pushing for more natural products, as some of the additives used in food processing and preservation have raised health concerns (Winkler et al., 2016; Younes et al., 2018; Partridge et al., 2019) and triggered negative consumer perception (Buchler et al., 2010; Bearth et al., 2014; Bärebring et al., 2020; Jansen et al., 2020).

### Functional Food Ingredients and Health

Lifestyle-associated complex diseases, especially metabolic conditions such as diabetes and obesity (Baboota et al., 2013; Rebello et al., 2014; Gentile et al., 2018), do not conform to the traditional “one disease one drug target” paradigm (Hopkins 2008). Moreover, these conditions are typically chronic (Bailes 2002; Bray, Kim et al., 2017), and are amenable to dietary therapy (Barnard et al., 2009; Sami et al., 2017; Zou et al., 2018; Castellana et al., 2019; Estruch and Ros 2020). They cannot be sustainably addressed by pharmaceutical means only, i.e., through “repair after onset”; rather, prevention and nutritional intervention are necessary complementary strategies to alleviate the public health burden of lifestyle-associated chronic disease (Mattei et al., 2015; S.; Sharma et al., 2010; Turner-McGrievy et al., 2017; Schwarz et al., 2008). Large-scale clinical studies repeatedly demonstrate that foods affect the risk of incidence and mortality from common chronic diseases that are rapidly rising in society (L. Wang et al., 2018; De Koning et al., 2012; Kenfield et al., 2014; Tasevska et al., 2014; Salas-Salvadó et al., 2014; Esposito et al., 2017; Dicker et al., 2018; Muraki et al., 2013), thus bearing the potential to improve global population health in a cost-effective manner (Eussen et al., 2011; Yang et al., 2018; J.; Wang et al., 2020). For example, FFIs can mitigate the effects of chronic, unresolved inflammation (Rein et al., 2019; K.; Kennedy et al., 2020a) that can cause damage to healthy cells, tissues and organs, and FFIs can actually maintain and restore homeostatic balance (Stampfer et al., 2000; Muraki et al., 2013; Valls-Pedret et al., 2015; Alkhatib et al., 2017). While pharmacological compounds typically act on disease states after onset, FFIs exert multiple, subtle, long-term effects in a concerted fashion that delay or prevent such disease states. The benefits of FFIs become evident upon chronic consumption of a blend of bioactives, where the (food) matrix matters for both bioavailability and bioefficacy (Kussmann et al., 2007; Holst and Williamson 2008).

So which factors are impeding a more rapid and systematic progress in translational nutrition studies for health maintenance and disease prevention?

a) Study design: One important explanation is inconclusive and at times contradictory scientific findings as to the health effects of various functional foods and ingredients (Ferreira et al., 2016; Hungin et al., 2018). These partly result from poor comparability of nutritional intervention studies, which are in turn due to lack of standards for diets and study designs (Kaput et al., 2015). Therefore, standardised studies performed across different ethnicities with biochemically defined diets and ingredients are required (Kaput et al., 2015).
b) FFIs discovery and characterisation: a further key limitation in translational nutritional studies is the incomplete biochemical and biological characterisation of active ingredients and the serendipitous discovery process for natural ingredients with health benefits.

This second issue can be effectively addressed with the adoption of AI into the discovery and characterisation pipeline which is the focus of this paper.

### Characterisation of Functional Food Ingredients—For a Healthier and More Sustainable Food Chain

Customarily, characterisation comes after the identification of a FFI and encompasses the determination and description of biochemical, biophysical, and biological properties of the active molecule(s) with all of these, and especially the latter, allowing for assignment of health benefits to FFIs. Such characterisation is usually performed with bioanalytical methods in the “wet laboratory” and provided information about key properties of FFIs when administered as a dietary component or as a supplement. These properties concern safety profile, stability after (oral) ingestion and across gastro-intestinal digestion, amenability to active or passive transport from the gut lumen across the brush border membrane into the blood stream (systemic bioavailability), and availability in the target tissue (local bioavailability). Ultimately, this information provides a comprehensive understanding of mechanism-of-action, overall bioavailability, bioefficacy and therefore dosage of FFIs. This fundamental understanding of the ingredient’s molecular properties and functions informs on:

- Reproducible analytical and production methods.
-Application of the ingredient within a diet or as a supplement.
- An efficient route to clinical trials.
- Regulatory requirements.
- Further scientific research on the ingredient’s health effects.

In addition to the discovery and validation of new health-beneficial FFIs or the assignment of new benefits to existing FFIs, the above described systematic characterisation also enables many of the risk-associated food product additives to be replaced by natural food-derived compounds, in particular peptides, resulting in a safer food chain (Scotter 2011; Schweiggert 2018). Such food additives typically confer stability for longer shelf-life, and sensorial attributes like flavour, texture, and colour to the food product (Parmar, Angad, and Gupta Phutela 2015; Solymosi et al., 2015; Taghvaei, 2015). Both a strong consumer demand and compelling scientific reasons have triggered the replacement of artificial, synthetic food additives by natural, typically plant-derived, alternatives and, thereby, reduced the necessity for potentially harmful food additives that are typically the source of consumer concern (Van Gunst and Roodenburg, 2019; Poortvliet and Hartemink, 2013).

Given the obvious scientific advantages and consumer needs, why has FFI characterisation science not progressed at speed? Notably, there are a number of challenges in characterising FFIs.

First, from an analytical perspective, characterisation of bioactive food ingredients requires expensive and specialized instrumentation and methods to identify, quantify and biochemically characterise FFIs and, therefore, quality control standards need to be established. Numerous compound-specific bioanalytical methods and assays have been developed for the micronutrient classes of vitamins (Mitić et al., 2011; Nannapaneni et al., 2017; Y.; Zhang et al., 2019), essential fatty (Ichihara et al., 1996; Birjandi et al., 2014; Dołowy and Pyka, 2015) and amino acids (Fonseca et al., 2018; Virág et al., 2020) and minerals (trace elements) (Carapelli et al., 2020; Tempesta et al., 2020). While those methods have proven to be precise, they typically represent “one-off” methods, and are neither versatile nor high-throughput (Höller et al., 2018). These traditional methods typically rely on multi-step fractionation, separation and isolation of bioactive ingredients via size exclusion (X. Wang et al., 2017; Gallego et al., 2018), ion exchange (Mukherjee et al., 2016; Acquah et al., 2019), or reversed-phase high-performance liquid chromatography (Bah et al., 2016; Liu et al., 2018), coupled to compound detection, typically by ultraviolet (UV) absorption (Brodbelt, 2016). Due to the lack of compound-specific detection when using UV, the final confirmation of bioactive compound identity relies on the employment of internal or external standards, adding cost and time to each analysis (Schnatbaum et al., 2020). Mass spectrometry coupled with liquid chromatography (LC-MS/MS) has replaced or is replacing most of those methods because of its universal applicability to virtually any kind of biochemical compound, the possibility of analyte multiplexing in one run, speed of operation, and the capability of *de novo* identification and structure elucidation of biochemical compounds, largely enabled by MS/MS fragmentation of the molecule of interest (Tribalat et al., 2006). LC-MS/MS is particularly suited for the analysis of bioactive food peptides, because of its high-throughput capability of sequencing and quantifying peptides (Bronsema et al., 2013; “Simplification of Complex Peptide Mixtures for Proteomic Analysis: Reversible Biotinylation of Cysteinyl Peptides—Spahr et al., 2000—ELECTROPHORESIS—Wiley Online Library,” n.d.). Hence, peptidomics, and more specifically food peptidomics, have evolved within the overarching mother discipline of proteomics as the platform for comprehensive characterisation of the protein and peptide complement in both food raw material and products (Panchaud et al., 2012). Similarly, high-throughput and paralleled LC-MS/MS methods have recently been developed for the comprehensive profiling of essential nutrients such as vitamins (Nagy et al., 2007), essential fatty (Hewawasam et al., 2017; Serafim et al., 2019) and amino acids (Le et al., 2014; Kambhampati et al., 2019). The task of identifying and quantifying minerals (or trace elements) as a further complement of essential nutrients, requires even more sophisticated analysis with inductively-coupled plasma mass spectrometry having more recently been established as the state-of-the-art for such purpose (Konz et al., 2017).

Second, from a biochemical and biological perspective, the molecular food characterisation process involves the disentanglement of millions of compounds present at different concentrations in the natural source, which essentially makes the process akin to space exploration at molecular level. For example, curcumin is a polyphenol found in Curcuma species. Such species are considered functional food that exhibit moderate anti-oxidant and anti-inflammatory effects *in vitro* and yet, due to the complexity of the curcuma compound class, individual molecules responsible for these activities have not yet been identified (Schaffer et al., 2015; Hewlings and Douglas, 2017). Conversely, even if a potential bioactive within a functional ingredient is identified, assessing stability and bioavailability, attributing functionality, and validating effects *in vitro*/*in vivo* has proven to be difficult (Corrochano et al., 2019; Feng et al., 2019). The latter challenge of assigning a specific effect to a molecule is characteristic for food bioactives, because—unlike pharmaceutical compounds—food bioactives exert multiple, subtle, long-term effects in a concerted fashion, rather than a single, strong, immediate effect conferred by a single molecule (Cal et al., 2020; Corrochano et al., 2021). In addition, the food matrix adds to the mode-of-action of food bioactives (Udenigwe and Fogliano, 2017; Sun et al., 2020). Consequently, the attempt to isolate both the active principle and its effect “in the same fraction” often fails because the “purer” the compound the smaller the effect (Papetti 2012).

### Traditional Ingredient Discovery and Characterisation

Health-augmenting bioactive components of natural products have traditionally been discovered serendipitously, in an *ad hoc*, hazardous fashion. For example, the discovery and establishment of essential nutrients such as vitamins (from “vital amines”, i.e. initially thought of as amine molecules essential to sustain life) took decades over the 20th century (DeLuca 2016; Jones, 2018; Cerullo et al., 2020) and, in turn, excessive financial resources had to be invested to proceed from discovery *via* validation to production (Acevedo-Rocha et al., 2019). Often, the absence of an essential nutrient has been observed to be associated with a disease and this disease has then been causally related to a deficiency syndrome. For example, the high occurrence of scurvy among sailing seafarers could be attributed to lack of vitamin C over long periods of time, which was due to the absence of fruits from their diets (Magiorkinis et al.2011). Conversely, the presence of certain dietary components has been correlated with health benefits. For example, Mediterranean diets are typically rich in olive oil and, therefore, in monounsaturated fatty acids, and a causal relationship with reduced incidence of cardiovascular disease in Mediterranean populations has been postulated (Lorgeril and Salen, 2007; Massaro et al., 2010; De). Notably, many of these correlational observations have to date still not been solidified into causality between nutrient (un)availability and health/disease condition—simply because of lacking directional relationships between biochemically characterised diets and bioactive ingredients on the one hand and health outcomes in humans on the other hand (Kussmann et al., 2007). Another, much more recent example of an initially serendipitous functional ingredient discovery, is human milk oligosaccharides (HMO) that were first discovered ∼1930 when a human milk carbohydrate fraction named “gynolactose” was identified [the discovery process is reviewed in Bode (2012)]. This discovery established the foundation for a century of research (Kunz, 2012), but it is only in recent years that this effort has delivered decisive data describing the benefits of HMOs in the modification of intestinal microbiota (LoCascio et al., 2007; Asakuma et al., 2011; Hoeflinger et al., 2015; Garrido et al., 2016), the anti-adhesive effects against pathogens (Idänpään-Heikkilä et al., 1997; Ruiz-Palacios et al., 2003; Angeloni et al., 2005; Morrow et al., 2005; Yu et al., 2016), the modulation of the intestinal epithelial cell response (Kuntz et al., 2009; Holscher et al., 2014), and the development of the immune system (Castillo-Courtade et al., 2015; Goehring et al., 2016; He et al., 2016; Comstock et al., 2017; Donovan and Comstock 2017; Dicker et al., 2018). In turn, driven by increased consumer interest in improving gut health and in the consumption of health-promoting dietary supplements (“Human Milk Oligosaccharides Market Size | Industry Report, 2027, 2021”), the emerging global HMO market value is expected to increase five-fold in the present decade (“Global Human Milk Oligosaccharides Market Expected to Grow with a CAGR of 21% During the Forecast Period, 2018–2027—ResearchAndMarkets.Com | Business Wire, 2020”). Further exponential growth in the HMO market is not being hindered by lack of demand, but limited primarily by lack of technology for large-scale production, in addition to high R&D cost and stringent government regulations (“Human Milk Oligosaccharides Market Size | Industry Report, 2027, 2021”).

These examples of serendipitous ingredient discovery demonstrate the urgent need for a more efficient technology-aided functional food ingredient characterisation pipeline. As discussed above, the first big technological revolution in bioactive compound—and also functional ingredient—discovery and characterisation came with the advent of high-throughput (HTP) screening. With the richness of biofunctionalities of compounds present in the natural product and food space and the resulting potential for therapeutic effects in mind, these platforms were built to enable high-throughput fractionation, identification, and quantification of (expected to be novel) natural bioactives. The development was catalysed by a combination of technological breakthroughs in miniaturised and multiplexed separation (Chu et al., 2006), automated liquid handling (robotics) (Chow et al., 2018), more sensitive and versatile detection techniques, such as MS, and advanced bioinformatics and in silico techniques to interpret MS data (Li-Chan and Eunice, 2015). Despite notable successes, especially in drug discovery (Chan et al., 2010; Takebe et al., 2018), the yield of new bioactive food compounds and functional ingredients has been limited, because of the particular challenge with food bioactives acting together and within the food matrix, as discussed above.

**FIGURE 1:**
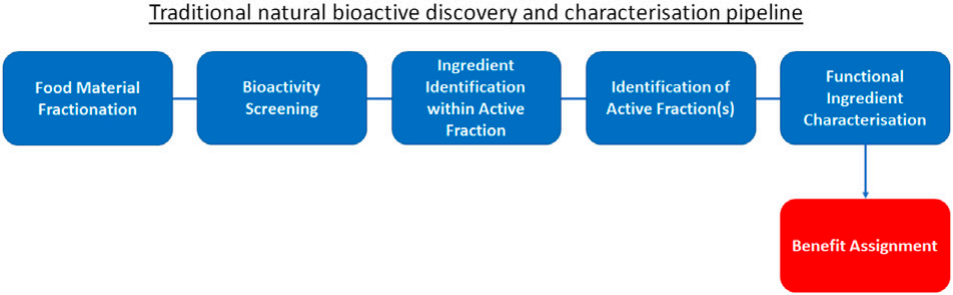
Traditional natural bioactive discovery and characterisation pipeline. Traditional process (blue) to discovery and characterisation of functional ingredients. This process begins with food material fractionation, followed by bioactivity screening, identification of active fraction(s), ingredient identification within active fraction and functional ingredient characterisation. Finally, the benefit is assigned to the bioactive (red) based on the screening results.

The integration of these new “wet laboratory” technologies such as mass spectrometry and HTP screening into traditional ingredient discovery and characterisation has certainly advanced the field (Figure 1). However, due to still high costs for equipment and analysis execution, this traditional approach of “food material fractionation → bioactivity screening → identification of active fraction(s) → ingredient identification within active fraction → functional ingredient characterisation → benefit assignment” has not delivered on rapid expansion of the pool of truly novel, disease-modifying and health-maintaining molecules available to consumers: it is simply not viable to experimentally unravel and confirm the effect of all molecules within natural products for multiple therapeutic benefits. Moreover, employing this traditional approach, health claims are assigned only after bioactivity has been established: this approach does not start with a health benefit and consumer need to be addressed, rather it searches for an application once the fortuitous new activities of the bioactives have been identified. As a result, to date only a limited number of bioactive ingredients with true life-changing properties, such as the essential micronutrients vitamins (Jacobson and Jacobson, 2018; Maqbool and Aslam, 2018), omega-3 and -6 fatty acids (Thomas et al., 2009; Watanabe and Tatsuno, 2017; Shahidi and Ambigaipalan, 2018) or phytonutrients such as polyphenols (Nagy et al., 2009) and anthocyanins (Speer et al., 2020; Tena et al., 2020), with the latter two not being classified as “nutrients”, have been characterised and successfully marketed. In view of this, identifying a malleable target in relation to a health claim may be a more beneficial approach to discovery, and one where advanced computational techniques offer the greatest opportunity.

## Artificial Intelligence in Functional Food Ingredient Characterisation

### Advancing From Classical Bioinformatics to Predicting Novel Bioactives

**FIGURE 2:**
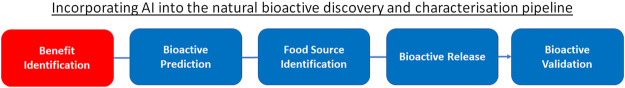
Integrating AI into the natural bioactive discovery and characterisation pipeline. Specific health benefits or replacement needs (red) are targeted at the beginning of the process. AI integration allows for prediction of bioactives with pre-specified health benefits and subsequent selection of a most suitable source, which contains an abundance of these bioactives, plus bioactivity confirmation (blue).

As discussed in *Traditional Ingredient Discovery and Characterisation*, combining *in silico* bioinformatics, *in vitro*, and HTP screening approaches has greatly advanced the characterisation of FFIs (Li-Chan and Eunice, 2015). For example, bioinformatic tools that implement sequence similarity searches, such as the Basic Local Alignment Search Tool (BLAST) (Altschul et al., 1990) can be used to in silico mine the proteomes that contain specific proteins of interest, and to identify molecular pathways, functions and diseases in which these proteins are involved (Kanehisa and Goto, 2000; Harris et al., 2004; Carvalho-Silva et al., 2019). However, these processes typically analyse and extrapolate from known data and cannot *de novo* predict molecular entities and functions. Such *de novo* computational identification of bioactives and their functions could and should be expedited by the incorporation of artificial intelligence (AI) methods, in particular deep learning approaches, providing true innovation in areas such as functional food and small molecules at an unprecedented rate, resulting in novel insights for a range of questions (Kaur and Kumari 2019; Morley et al., 2020; Zorn et al., 2021; A. K.; Sharma et al., 2017; Kathy; Kennedy et al., 2020b; K.; Kennedy et al., 2020a; Chauhan et al., 2021; Corrochano et al., 2021; Cal et al., 2020). With a focus on peptides, we argue here for complementing, if not replacing, the traditional sequence of FFI characterisation (Figure 1) with an AI-powered alternative (“benefit definition → bioactive prediction → food source identification → bioactive release → bioactive validation”; Figure 2) by placing artificial intelligence upfront in the entire process. In other words: AI changes the paradigm from screening and retrospective benefit assignment to design according to predefined benefit.

### Advancing From Screening to Design: The AI-Powered Discovery and Subsequent Validation Workflow

An ideal starting point and guiding principle to FFI characterisation that would address the current *ad hoc* discovery pipeline limitations would be to first understand and define the nutritionally actionable health needs of consumers and food chain-relevant replacement requirements. This should be followed by design and development of a commercially viable approach to identify, source, unlock and characterise a safe bioactive food peptide solution that is specific and effective and addresses needs in a cost-effective manner. For an integrated AI workflow for ingredient discovery and characterisation, once a health benefit is targeted, data in the public domain are curated from structured and unstructured data sources; these sources include scientific literature, patents and public databases that are searched for known peptide sequences with relevant bioactivity. Of note, the increased availability of food and plant molecular data has resulted in the improvement of relevant curated datasets (Westerman et al., 2020; Jensen et al., 2015; Rutz et al., 2021). These data are combined with information collected from different mass spectrometry-based peptidomics studies of plant sources and FFIs (discussed in *AI Integration With Peptidomics*) and in-house bioactivity validation data (discussed in *Circular Science: The Iterative Feedback Loop*). All these data are manually curated to ensure robust standards required for building training datasets. Typically, training datasets are compiled consisting of positive bioactive datasets, where peptide sequences have been shown to be efficacious for a specific activity either in published literature, databases or in-house assays, as well as negative datasets, where a peptide sequence did not exhibit the specified bioactivity (Kurczab et al., 2014). If there are only positive datasets available, random sequences may be used as the negative component for training purposes. The positive and negative datasets are used as input to build the algorithmic architecture that will predict peptides for a given bioactivity. These datasets essentially “train” the predictive architecture, in an iterative process, which results in improved accuracy of peptide prediction.

### AI Integration With Peptidomics

The power of AI-guided discovery and characterisation becomes particularly evident in the case of peptide-containing food protein hydrolysates, which represent a large source of FFIs with virtually unlimited benefit potential. Protein hydrolysates are widely available in the food market, for example as infant formulae based on cow’s milk; soy, or rice protein hydrolysates; and fermented foods such as yoghurt or kefir (Schaafsma, 2009). They contain large complex networks of interacting bioactive peptides with attributable health benefits (Hernández-Ledesma et al., 2014; Nasri 2017). To untangle these networks and characterise key bioactives and functions within, an integrated peptidomics-AI platform has recently been proven to be successful by our laboratories (Corrochano et al., 2021; Casey et al., 2021; Kathy Kennedy et al., 2020b; K. Kennedy et al., 2020a; Chauhan et al., 2021). As previously discussed, mass spectrometry-based peptidomics of food protein hydrolysates is a key input to the AI platform: the process begins with LC-MS/MS-based peptidomics that identifies and quantifies peptides in their native state, as released by mild hydrolysis from parent proteins in the natural food source, which is not limited to a single type of food or plant source. Physicochemical characteristics such as molecular weight, charge, length, and hydrophobicity can be assigned to these peptides, thus creating libraries of invaluable parsed data from a wide range of sources, including food-by products, ready to be leveraged by the AI architecture, as described in *Advancing From Classical Bioinformatics to Predicting Novel Bioactives*. This approach creates millions of entries from natural source-derived in-house peptidomic data, which are then combined with the manually curated peptide data sourced from the public domain, and results from subsequent in-house validation of peptide efficacy in relevant bioactivity assays. Leveraging this data pool, these peptide libraries can be classified into various structural and functional categories using deep learning approaches, parameters such as toxicity, solubility, size, polarity and binding dynamics and many less definable albeit important characteristics are inferred from the AI architecture. Ultimately, any number of these different deep learning approaches are chosen depending on their relevance to the area of interest and have been shown to be successful in the identification of peptides with anti-microbial (Mohan et al., 2019), anti-aging (Kathy Kennedy et al., 2020b; Kathy Kennedy et al., 2020a), or anti-inflammatory (Rein et al., 2019) activity.

### Circular Science: The Iterative Feedback Loop

**FIGURE 3:**
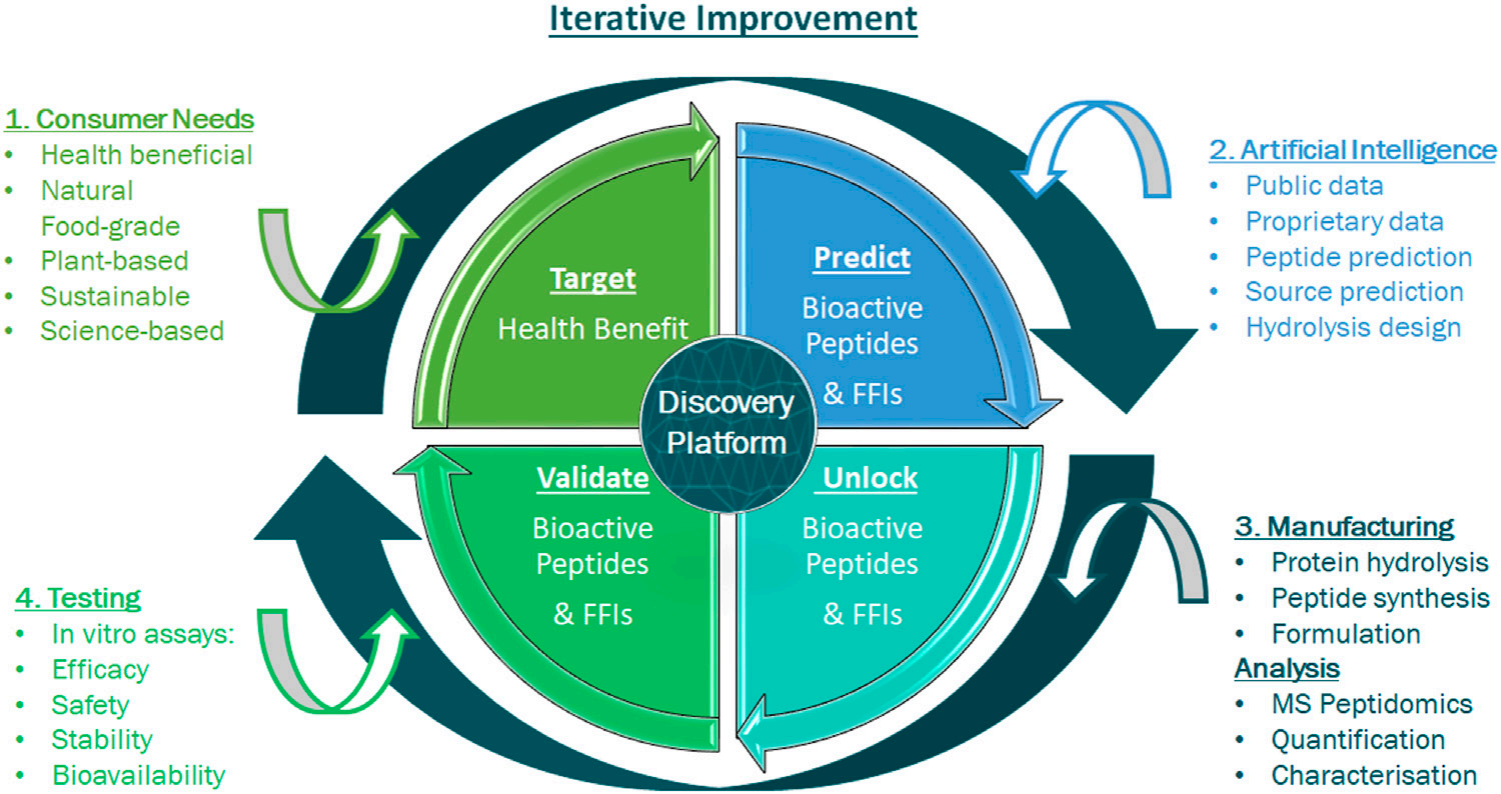
Iterative feedback loop for an integrated discovery and validation of efficacy platform. This consists of different technologies to explore the molecular diversity in plants and foods and create characterised natural, safe and effective health promoting bioactives for consumers. A number of processes are integrated in creating and characterising a functional food ingredient. 1) A consumer-driven health benefit is targeted. 2) The Artificial Intelligence (AI) process begins with data curation, predicts key bioactives and selects preferred ingredients. 3) Predicted bioactive peptides and ingredients are unlocked and produced. LC-MS/MS based-peptidomics identifies and characterises the ingredients’ peptide profile. 4) Predicted peptides and ingredients are assessed for relevant bioactivity, safety and toxicity, as well as for bioavailability using simulated gastric intestinal digestion (SGID) and investigation of stability in a relevant human matrix, i.e. blood plasma. The output of this integrated discovery platform is a characterised functional food ingredient that addresses an unmet health benefit.

In addition to the hydrolysate-derived and peptidomics-analysed peptide networks, individual predicted bioactive peptides are synthesised chemically and validated for efficacy *in vitro,* thereby elucidating mechanisms-of-action and creating a sophisticated real-time feedback loop of positive and negative data (Figure 3). This feedback constantly refines the deep learning algorithms resulting in ever-improving accuracy of bioactive peptide prediction. Once positive bioactive peptides are identified, AI is used to search natural plant and food proteomes for the presence of those positive peptides within the protein complement of a plant or food source. Once such a suitable source proteome is identified, AI informs the design of the enzymatic hydrolysis process (selection of food-grade enzymes) to be applied to unlock the targeted peptide and generate a hydrolysate with the desired peptide profile. Like the predicted and synthesised individual peptides, produced FFIs are also validated for efficacy *in vitro*, creating more valuable positive and negative data feedback for the AI platform. In addition to bioactivity feedback, the safety and toxicity of predicted peptides and FFIs as well as peptide stability across oral ingestion and gastro-intestinal digestion are also assessed, using cell viability assays (Cal et al., 2020) and *in vitro* digestion models (Corrochano et al., 2021) that are incorporated as part of the iterative learning process (Figure 3). Resulting from this AI approach, the end product FFI exhibits high bioefficacy, has a good safety profile and is characterised with a pre-approved list of key constituent peptides that have been synthesised and validated *in vitro* for a predicted activity.

For building an ingredient dossier and substantiating health claims, it is not only important to characterise key bioactivities of ingredients within a FFI but to also assess the ingredient’s stability across the gastro-intestinal digestion, its transport properties from the gut lumen to blood plasma, and its bioavailability at systemic and target tissue level. Of note, current AI architecture has the ability to predict peptides with gastro-intestinal resistance (Corrochano et al., 2021). Therefore, further simulated gastrointestinal digestion and stability experiments *in vitro* and *ex vivo* using FFIs bears the potential for future AI applications to identify key latent features, and therefore, predict the bioavailability profile of bioactive peptides and FFIs.

## Case studies of AI-powered natural bioactive peptide discovery and characterisation

### Anti-Inflammatory FFI

An example of a successful AI-powered benefit-driven bioactive FFI discovery is the identification and characterisation of novel anti-inflammatory bioactive peptides to address chronic low-grade inflammation. This study applied an ensemble of deep learning models to unstructured (publications, patents) and structured (data repositories) public data, as well as to proprietary peptidomic data, molecular docking simulations and phenotypic data accrued from internal experimental screening, to predict novel peptides with immunomodulatory potential from a large input set of peptide sequences from plant origins. Using an untargeted predictive approach, Asian rice was shown to be a candidate source that contained the novel predicted immunomodulatory peptides encrypted in Asian rice proteins. A hydrolysate was designed from the Asian rice bulk protein complement to create a novel FFI, rice Natural Peptide Network (NPN) (Rein et al., 2019). This FFI contained seven key constituent bioactive peptides that were physicochemically characterised and shown to exert immuno-modulatory effects *in vitro*. In proof-of-principle human feeding trial and a kinetic human trial, rice NPN reduced circulating TNF-α and improved physical performance in a series of challenges (such as a chair stand test) in an elderly population showing “inflammaging”, i.e. immune-senescence (K. Kennedy et al., 2020a; Rein et al., 2019). Of note, in this case of rice NPN, the time from discovery to commercial launch was approximately 2 years (Nuritas 2018), highlighting the speed at which a consumer need can be addressed using an AI-driven bioactive food peptide discovery and characterisation process.

### FFI for Muscle Health

Complementary to starting with a targeted health benefit, (“from benefit to bioactive”), AI can be leveraged to characterise an already known FFI or plant/food source in terms of constituent bioactives with a targeted functionality (“from source to benefit”). This approach was adopted to characterise a hydrolysate derived from *Vicia faba*, NPN_1, that was previously identified to prevent muscle atrophy *in vivo* (Cal et al., 2020). Here, two constituent peptides were predicted to increase protein synthesis and decrease inflammation and subsequently showed positive *in vitro* effects on ribosomal protein (S6) phosphorylation and reduction of TNF-α, respectively (see Corrochano et al., 2021 for in depth detail on predictive architecture methods utilised) (Corrochano et al., 2021). S6 phosphorylation induces the translation of mRNA transcripts for ribosomal proteins and elongation factors that induce muscle protein synthesis (Peterson and Schreiber, 1998; Gordon et al., 2013), while TNF-α is responsible for producing chronic inflammation, which is implicated in skeletal muscle dysfunction (Londhe and Guttridge, 2015). As discussed in *Characterisation of Functional Food Ingredients—For a Healthier and More Sustainable Food Chain*, assessing the bioavailability of an FFI within a diet is key to characterising the bioefficacy of the ingredient. Considering this, Corrochano et al. (2021) demonstrated *in vitro* that both of the peptides with predicted activity within NPN_1 from *Vicia faba* survived simulated gastrointestinal digestion, were transported across the intestinal barrier, and exhibited notable stability in human plasma (Corrochano et al., 2021).

These two examples illustrate the game-changing impact of AI in discovery and characterisation of peptide-based FFIs. When integrated into the process, AI can:

- Extend the discovery space to the entire plant and food kingdom because peptides are the largest class of genome-encoded nutrients and are therefore amenable to prediction of function and source.
- Direct the discovery process according to predefined, unmet consumer needs, be they health care- or food chain-related.
- Accelerate the discovery and subsequent validation process by rapid development and improvement of predictors and an efficient prediction-experiment feedback loop.
- Reduce the number of candidates to be characterised biochemically and biologically.
- Inform on clinical trial design and FFI health claims.
- Efficiently build a peptide-based FFI knowledge base that informs both producers and consumers, fuelling food innovation and augmenting science-backed nutritional health benefits.

## Conclusion

Given the widespread consumer acceptance of technology as a facet of their healthcare regime and the potential importance of FFI in preventive and therapeutic interventions for many chronic disorders, it is becoming clear that we are standing on the precipice of the next age of nutrition technology. The vision of health care, enabled by both nutrition and medicine (with an increasing overlap between the two) is changing from being disease-focussed in a medical setting, to a prevention-oriented practice, guided by health knowledge-empowered consumers. Ultimately, what does this next age of nutrition technology look like for the consumer? At present, most FFIs are developed in extrapolation from a limited number of ingredients with known health effects, in an inefficient, unfocussed, costly, and exceptionally slow manner. There is a vast reservoir of health-promoting and disease-defeating molecules in nature and the use of AI will enable the most efficient discovery and characterisation of innovative physiochemically and biologically characterised FFI products. These nutritional interventions will have the capability to be developed to address unmet consumer needs in the future at the levels of both individual and population health and a safe and sustainable food system. As food and nutrition become increasingly perceived as a knowledge-based health care and industry sector, we believe that integrating AI into FFIs discovery and development will greatly enhance the delivery of safe and cost-effective solutions for improved human and animal health as well as a safer and more sustainable food chain, over the decades to come.
